# Resection of the Upper Jaw, with Preservation of Portion of Hard Palate and Incisor Teeth

**Published:** 1875-04

**Authors:** 


### ARTICLE VIII.

### *Resection of the Upper Jaw, with Preservation of the Muco-periosteal Covering of the Hard Palate and Incisor Teeth.*

In the *Berliner Klinische Wochenschrift* for Nov. 3d, 1873, is reported a case in the clinic of Profeesor Linhart, in which he performed the above operation for the second time. The patient was a married woman, aged thirty-three. She went into a lucifer-match manufactory at the age of twelve, and, about a year afterwards, she seems to have become affected with match-makers' disease. On admission into the Royal Julius Hospital in May, 1873, her condition was as follows: There was an abscess in the lachrymal sac on the left side; and on examining the mouth, it was found that all the teeth of the upper jaw with the exception of the two last molars of the left side were loose and nearly falling out. Only the two incisors of the right side and the first of the left side were firm. The teeth of the lower jaw were complete and sound; a tooth had been extracted from the upper jaw some time previously (the carious tooth, which had been the means of the introduction of the disease,) and at this spot were two sinues, which discharged a thin, copious, and very fetid pus. A probe, which could be easily passed, detected dead bone. The gums and mucous membrane of the mouth were pale and inclined to bleed. The disease was diagnosed as phosphorous necrosis of both upper maxillæ. On May 18, Dr. Linhart performed the following operation: Chloroform having been administered the loose teeth, with the exception of the incisors and two last molars of the left side, were first removed. An incision was next made from between the eyebrows, perpendicularly downwards over the bridge of the nose, through the middle of the septum, and upper lip. The incision was carried as deep as possible, and the soft parts, with the periosteum of the upper jaw, on each side, were stripped off and reflected as far as the zygoma. Next an incision was made on the left side with an osteotome, in an oblique direction downwards from the apertura pyriformis, about half an inch below the infra-orbital margin, as far as the second molar tooth. On the right side the disease had spread wider; and the nasal process being divided, the osteotome was carried close under, and parallel with the infra orbital margin, as far as the greater wing of the sphenoid. The gum, with the three contained incisors and muco-periosteal covering of the hard palate, was now stripped off and turned down. The detached flap lay on the tongue, and served to conduct the blood out of the mouth and to prevent its going down the larynx. The bones were separated from their attachments and removed. The muco-periosteal covering of the hard palate was first fastened, and then the cheek-flaps brought together over the bridge of the nose. Following Bardeleben's method, the covering of the hard palate was joined within to the partially detached mucous membrane of the cheek; an armed needle was passed through its free edge, and out through the cheek on each side, and tied over a quilled suture. The external wound was brought together by twisted sutures and hare-lip pins. On the third day all the stitches were taken out, with the exception of those in the palate. The edges of the wound united by first intention, and the pus flowed out between the front opening of the newly formed palate and the upper lip. On the seventh day the sutures in the palate were removed, and it was found that the covering of the hard palate was completely united. It formed a concave bridge, which supported itself.

The three front teeth were not quite firmly set, but could be pressed level. This was effected by placing a piece of cork in the mouth. The gaps soon filled in with granulations, and the muco-periosteal flap and the teeth became rapidly firmer. On her discharge from the hospital on July 12, she could eat and drink, and spoke in a firm voice scarcely at all nasal.

[The great interest of the foregoing case is the preservation of the teeth; the operation of removal of both bones, either partial or entire, having been frequently performed in cases of phosphorus necrosis.]—*London Med. Record.*

---

